# A case of camrelizumab-induced anaphylaxis and successful rechallenge: a case report and literature review

**DOI:** 10.3389/fonc.2025.1537205

**Published:** 2025-03-21

**Authors:** Ping Song, Yuqi Jin, Lifang Dai, Lijun Fang, Yinuo Tan

**Affiliations:** ^1^ Department of Nursing, The Second Affiliated Hospital, Zhejiang University School of Medicine, Hangzhou, Zhejiang, China; ^2^ Department of Medical Oncology (Key Laboratory of Cancer Prevention and Intervention, China National Ministry of Education), The Second Affiliated Hospital, Zhejiang University School of Medicine, Hangzhou, Zhejiang, China; ^3^ Zhejiang Provincial Clinical Research Center for CANCER, Hangzhou, Zhejiang, China; ^4^ Cancer Center of Zhejiang University, Hangzhou, Zhejiang, China; ^5^ Department of Comprehensive Rehabilitation, Hangzhou Shangcheng District People’s Hospital, Hangzhou, Zhejiang, China

**Keywords:** immune checkpoint inhibitors, camrelizumab, anaphylaxis reaction, case report, anaphylaxis shock

## Abstract

Immune checkpoint inhibitors have been extensively utilized in the treatment of various malignancies, with camrelizumab being one of the agents in this therapeutic class. In this study, we report for the first time a case of an allergic reaction to camrelizumab in a patient with nasopharyngeal carcinoma, who was successfully rechallenged after antiallergic treatment. The patient, a 62-year-old male, was diagnosed with advanced nasopharyngeal carcinoma, exhibiting cancer infiltration and multiple metastases. He underwent multiple cycles of therapy, tolerating camrelizumab, nab-paclitaxel, and nedaplatin (200 mg of camrelizumab every 3 weeks) without adverse reactions in the first four cycles. However, during the fifth cycle, after the intravenous infusion of camrelizumab, he experienced gradual onset of dizziness and chest tightness within 15 minutes (peripheral arterial oxygen saturation was approximately 94%, blood pressure was 76/42 mmHg, heart rate was 83 beats per minute, and respiratory rate was 15 breaths per minute). The camrelizumab infusion was immediately halted, and the patient was treated with intravenous dexamethasone (10 mg) combined with intramuscular diphenhydramine, calcium gluconate, and 500 ml of normal saline; his blood pressure gradually increased to 110/80 mmHg within 10 minutes, and pruritic erythematous macules appeared on his skin, particularly on the upper limbs. Subsequently, nab-paclitaxel was infused, and upon completion, the erythematous macules on the limbs faded. The patient was then rechallenged with a slow infusion of camrelizumab, which was well-tolerated without discomfort or a drop in blood pressure. The patient did not report significant discomfort. Although acute allergic reactions are relatively rare among immune-related adverse events, due to the widespread clinical application of camrelizumab, its potential for allergic reactions should be given high priority.

## Introduction

With the continuous advancement of medical technology, the treatment of malignant tumors has evolved from traditional surgery, radiotherapy, and chemotherapy to more precise and personalized therapeutic approaches (1). In recent years, the emergence of immunotherapy has brought revolutionary changes to cancer treatment, particularly in certain types of tumors where it has become an integral part of the standard treatment regimen (2).

Immune checkpoint inhibitors (ICIs) reactivate the patient’s own immune system to combat tumors by blocking mechanisms that allow tumor cells to evade immune surveillance (3). Camrelizumab (SHR-1210) is an immune checkpoint inhibitor, a monoclonal antibody targeting the PD-1 receptor (4). It enhances the body’s antitumor immune response by blocking the interaction between PD-1 and its ligand PD-L1, thereby relieving the immunosuppressive effect of tumor cells on T-cells. Camrelizumab has been approved for the treatment of various malignancies, including esophageal squamous cell carcinoma and non-small cell lung cancer (5, 6).

Despite the significant potential of immune checkpoint inhibitors in oncology, they can also lead to adverse reactions, including immune-related adverse events. Anaphylactic shock is one of the severe adverse reactions, which, although rare, poses a threat to patients’ life safety when it occurs (7). In this article, we report a case of acute allergic reaction induced by the infusion of camrelizumab, and the successful rechallenge with camrelizumab after antiallergic management.

## Case presentation

### General information

The patient is a 62-year-old male who was diagnosed with nasopharyngeal carcinoma six years ago and has had stable disease control after multiple cycles of chemotherapy combined with radiotherapy. The patient has a smoking history of over 20 years, with 20 cigarettes per day, and a history of moderate alcohol consumption.

### Treatment course

**Figure 1:**
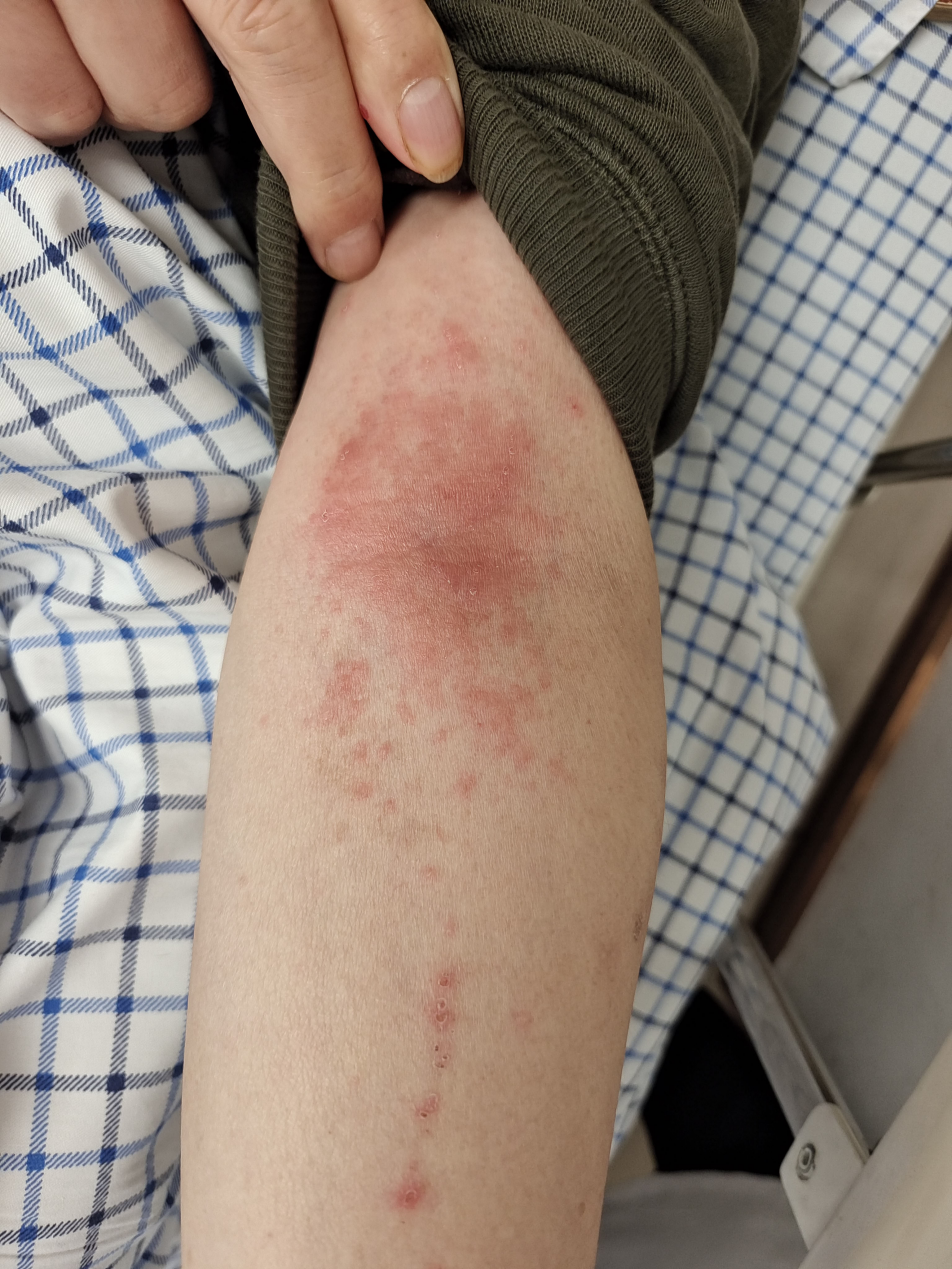
Allergic reaction following drug injection. The image shows erythematous rash and localized swelling on the forearm of the patient after drug administration, indicative of an allergic response.

The patient, a 62-year-old male, presented to our hospital’s Otolaryngology Department in 2018 with nasal congestion and blood-tinged nasal discharge. A biopsy of a nasopharyngeal neoplasm indicated non-keratinizing carcinoma, leading to a diagnosis of nasopharyngeal carcinoma. He underwent chemoradiotherapy with three cycles of the TP regimen, specifically consisting of paclitaxel liposome (Lipusu) 210mg on day 1 and nedaplatin 130mg on day 1, supplemented with antiemetic, gastric protection, and fluid support treatments. The radiotherapy concluded on November 2, 2018. The patient tested negative for Epstein-Barr virus and had regular follow-ups. A nasopharyngeal MRI in June 2024 showed progression of bone destruction compared to previous scans. A whole-body PET/CT imaging on June 29, 2024, revealed: 1. Mildly increased glucose metabolism in the right posterior wall of the nasopharynx post-radiotherapy for nasopharyngeal carcinoma, suggesting possible inflammatory changes or viable tumor tissue, with recommendations for follow-up; destruction of the pterygoid and abnormally increased glucose metabolism in the clivus region of the occipital bone, indicating possible tumor involvement. On July 17, 2024, August 8, 2024, August 30, 2024, and October 5, 2024, the patient received nab-paclitaxel 400mg intravenous infusion and nedaplatin 135mg intravenous infusion chemotherapy along with camrelizumab 200mg intravenous infusion for immunotherapy. On October 28, 2024, the patient was readmitted with a blood pressure of 134/85 mmHg. Fifteen minutes after the infusion of camrelizumab, he gradually developed dizziness and chest tightness (peripheral arterial oxygen saturation was approximately 94%, blood pressure was 76/42 mmHg, heart rate was 83 beats per minute, and respiratory rate was 15 breaths per minute), with clear consciousness. Suspecting anaphylactic shock, camrelizumab was immediately discontinued, and the patient was treated with intravenous dexamethasone (10 mg) combined with intramuscular diphenhydramine, calcium gluconate, and 500 ml of normal saline; within 10 minutes, his blood pressure gradually increased to 110/80 mmHg, and pruritic erythematous macules appeared on his skin, especially on the upper limbs (as shown in **Figure 1**). Subsequently, nab-paclitaxel was infused, and after completion, the erythematous macules on the limbs faded. After thorough communication with the patient and his family, who considered the drug to be expensive but effective and wished to try the remaining medication again, the patient was informed of the potential risks of recurrent anaphylactic shock and other life-threatening risks. The patient acknowledged the risks and was willing to accept them. The patient was then rechallenged with a slow infusion of camrelizumab at a rate of 10 drops per minute, with close monitoring of blood pressure, oxygen saturation, and other vital signs. The infusion proceeded smoothly without discomfort or a drop in blood pressure. The infusion rate was gradually increased to 30 drops per minute half an hour later, and the entire process was uneventful without adverse reactions. On December 13, 2024, January 3, 2025 and January 25, 2025, the patient received tislelizumab (0.2 g, D1) as part of immunotherapy maintenance, accompanied by anti-allergic prophylaxis with promethazine (25 mg), dexamethasone (5 mg), and loratadine (8.8 mg). The treatment was well tolerated, with no significant adverse reactions reported. Subsequent follow-up nasopharyngeal MRI indicated a reduction in the size of the skull base lesion.

### Literature search

The search terms “camrelizumab,” “immune checkpoint inhibitors,” and “allergic reactions” were used to retrieve relevant case reports from the Wanfang Data, Web of Science, and PubMed databases up to November 2024. An analysis and summary of the clinical characteristics and outcomes of the detected cases were conducted. As of November 2024, 4 similar case reports were identified from PubMed, and the summarized information is presented in **Table 1**.

**Table 1:** Literature review of infusion reaction/anaphylaxis caused by immune checkpoint inhibitor.

Author	Immune checkpoint inhibitor	Types of cancer	Adverse reaction	Occurrence time	Clinical treatment	
Choi B et al	Nivolumab	hepatocellular carcinoma	1. Facial flushing, shortness of breath, and low back pain 2. Allergy-like symptoms	1. 10 min after the second infusion 2. 11min after the third cycle of treatment	1. Discontinued and treated with diphenhydramine and hydrocortisone, followed by a slow infusion of the remaining nivolumab 2. Resolved with symptomatic treatment, switched to pembrolizumab for subsequent immunotherapy	Choi B, McBride A, Scott AJ. Treatment with pembrolizumab after hypersensitivity reaction to nivolumab in a patient with hepatocellular carcinoma. Am J Health-syst Pharm: Ajhp: Off J Am Soc Health-syst Pharm. 2019 Oct 15;76(21):1749–52.
Ogawara D et al	Nivolumab	lung squamous cell carcinoma	Skin itching and flushing which quickly spread all over the body, and the blood oxygen saturation decreased from 97% to 92	15 min after the second infusion	The infusion was stopped immediately and oxygen inhalation, chlorpheniramine, and methylprednisolone were given	Ogawara D, Soda H, Ikehara S, Sumiyoshi M, Iwasaki K, Okuno D, et al. Nivolumab infusion reaction manifesting as plantar erythema and pulmonary infiltrate in a lung cancer patient. Thorac Cancer. 2017 Nov;8(6):706–9.
Mercedes Sáenz de Santa María García et al	Nivolumab	hepatocellular carcinoma	Facial flushing, rash, and eyelid edema	Near the end of the third cycle	The symptoms disappeared spontaneously about 45 min after withdrawal	Sáenz de Santa María García M, Noguerado-Mellado B, Rojas-Pérez-Ezquerra P, Prieto-García A, Bartolomé-Zavala B, Tornero P. First case of allergy to nivolumab. J Allergy Clin Immunol, Pract. 2017;5(4):1140–1.
Liu K et al	Camrelizumab	Esophageal squamous cell carcinoma	Palpitation, dyspnea and a feeling of death; the pulse rate in the indoor air was 70 beats/min, the blood pressure was 69 centimeters of 24 mm mercury, the respiratory rate was 28 beats/min, and the pulse oxygen saturation was 86%	10 min after the second infusion	intravenous infusion, epinephrine, dexamethasone sodium phosphate, calcium gluconate and norepinephrine	Liu K, Bao JF, Wang T, Yang H, Xu BP. Camrelizumab-induced anaphylactic shock in an esophageal squamous cell carcinoma patient: a case report and review of literature. World J Clin Cases. 2022 Jun 26;10(18):6198–204.
Yizhuo Zhao et al	atezolizumab	small cell lung cancer	anaphylactic shock, such as dyspnea, cold limbs, and loss of consciousness. A	three minutes after the second infusion	oxygen, epinephrine, dopamine, methylprednisolone,	Zhao Y, Peng W, Abbas M, Shi M, Tang Y, Wang L, et al. Anaphylactic shock in a small cell lung cancer patient receiving atezolizumab therapy: a rare but potentially fatal complication. Invest New Drugs. 2022 Feb;40(1):209–14.
Ji Hyun Oh et al	atezolizumab	hepatocellular carcinoma	facial flushing and generalized itching and soon lost consciousness with hypotension and an oxygen saturation of 90%.	5 minutes after starting the first cycle of atezolizumab	dexamethasone,chlorpheniramine and norepinephrine	Oh JH, Seo KI, Kim HK, Choi GS. Successful desensitization to atezolizumab-induced near-fatal anaphylaxis in patients with hepatocellular carcinoma: a case report and literature review. Asia Pac Allergy. 2024 Aug;14(3):139–42.
Weiting Liang et al	Cadonilimab	lung cancer	chest distress and shortness of breath.	15 min after the second infusion	diphenhydramine 20 mg, promethazine 25 mg, and compound sodium chloride 500 mL. Dexamethasone 5 mg	Hong DI, Madrigal-Burgaleta R, Banerji A, Castells M, Alvarez-Cuesta E. Controversies in allergy: chemotherapy reactions, desensitize, or delabel? J Allergy Clin Immunol, Pract. 2020 Oct;8(9):2907-2915.e1.
Weiting Liang et al	Cadonilimab	nasopharyngeal cancer	multiple red skin bumps on the trunk and pruritus	during the fifth infusion	intravenous infusion of dexamethasone 10 mg and esomeprazole 40 mg,	Agrawal S, Statkevich P, Bajaj G, Feng Y, Saeger S, Desai DD, et al. Evaluation of immunogenicity of nivolumab monotherapy and its clinical relevance in patients with metastatic solid tumors. J Clin Pharmacol. 2017 Mar;57(3):394–400.
Weiting Liang et al	Cadonilimab	squamous cell carcinoma of the cervix	chest distress, facial flushing, abdominal pain, vomiting, and profuse sweating	3 min after the first infusion	Dexamethasone 30 mg, diphenhydramine 20 mg, promethazine 50 mg, and compound sodium chloride 500 mL were administered	Isabwe GAC, de Las Vecillas Sanchez L, Castells M. Management of adverse reactions to biologic agents. Allergy Asthma Proc. 2017 Nov 1;38(6):409–18.
Weiting Liang et al	Cadonilimab	hepatocellular carcinoma	sweating, low BP (64/42 mmHg), flaked red rash and pruritus appeared on the arm, neck, and buttocks	during the third infusion	intramuscular injection of butyryl 30 mg, diphenhydramine and dexamethasone	Ramírez-Cruz S, Lucena-Campillo MA, Vila-Albelda C, Garrido-Arévalo M, De Agustín-Sierra L, García-Díaz B. Desensitization protocol to nivolumab without corticosteroid use in a kidney cancer patient. Farm Hosp: Organo Of Expr Cient Soc Esp Farm Hosp. 2020 Jul 1;44(4):182–3.
Weiting Liang et al	Cadonilimab	cervical cancer	redness and swelling in the face, numbness, and itching in the mouth, low BP (85/60 mmHg),	during the second infusion	Dexamethasone 10 mg, cimetidine 0.4 g, and promethazine 25 mg	
Weiting Liang et al	Cadonilimab	lung cancer*	systemic cold sweats and rapid breathing, BP was 92/ 61 mm Hg, and HR was 104 beats/min	during the second infusion	Dexamethasone 10 mg, cimetidine 0.2 g,diphenhydramine 20 mg	
Weiting Liang et al	Cadonilimab	adenoid cystic carcinoma	shivering with undetectable low BP	during the seventh infusion	dexamethasone 10 mg	

## Discussion

In the new era of immunotherapy, PD-1 inhibitors such as camrelizumab have demonstrated significant efficacy in the treatment of various tumors. However, the allergic reactions they cause, particularly anaphylactic shock, though rare, can pose a serious threat to patients’ lives when they do occur (8). In this case, the patient experienced anaphylactic shock during the treatment of nasopharyngeal carcinoma with camrelizumab. After timely rescue and antiallergic treatment, the patient was successfully rechallenged with camrelizumab, continued the treatment, and achieved a good therapeutic effect.

According to the literature review, anaphylactic shock caused by monoclonal antibody biologics typically occurs in the early stages of treatment, especially in the first few cycles. These reactions are characterized by rapid onset, involving the skin, mucous membranes, or both, such as generalized urticaria, itching, or flushing, as well as respiratory impairment (e.g., difficulty breathing, asthma-bronchospasm, wheezing, reduced peak expiratory flow, and hypoxemia). Additionally, blood pressure drop or end-organ dysfunction (e.g., muscle rigidity, syncope, incontinence) are also common clinical manifestations (8–10).

In managing anaphylactic shock, rapid recognition and timely treatment are crucial. According to the guidelines of the European Academy of Allergy and Clinical Immunology (EAACI) Multidisciplinary Taskforce, any of the above symptoms, when present, should raise a high suspicion of an allergic reaction (11). In this case, the patient exhibited symptoms rapidly after receiving camrelizumab treatment, consistent with the clinical presentation of an allergic reaction. The patient also experienced a significant drop in blood pressure, which allowed for the diagnosis of anaphylactic shock.

The possibility of reusing the drug:

The question of whether to reuse the drug after an anaphylactic shock event is a complex one. Generally, once an anaphylactic shock has occurred, it is not recommended to reuse the same drug for safety reasons. However, each case requires an individualized assessment, taking into account factors such as the patient’s tumor status, treatment response, and the severity of the allergic reaction. In this case, the patient gradually developed symptoms such as headache and chest tightness 15 minutes after the infusion, rather than an immediate rapid drop in blood pressure, and did not exhibit loss of consciousness. In the management of the symptoms, the drug was discontinued, and antiallergic medications were administered along with fluid resuscitation, after which the patient’s blood pressure recovered, symptoms were alleviated, and no medications such as epinephrine were used. We considered that the patient’s anaphylactic shock was not extremely urgent and rapidly progressive.

Subsequently, regarding the continuation of the remaining camrelizumab infusion, we communicated fully with the patient and their family. They believed that the drug was expensive but effective and wished to try the remaining medication again. We informed the patient and their family of the potential risks of recurrent anaphylactic shock and other life-threatening risks. The patient acknowledged the risks and was willing to accept them. The patient was then rechallenged with a slow infusion of camrelizumab at a rate of 10 drops per minute, with close monitoring of vital signs such as blood pressure and oxygen saturation. The infusion proceeded smoothly without discomfort or a drop in blood pressure. After half an hour, the infusion rate was gradually increased to 30 drops per minute, and the entire process was uneventful without adverse reactions.

In the new era of immunotherapy, PD-1 inhibitors such as camrelizumab have shown significant efficacy in the treatment of various tumors. However, allergic reactions they cause, particularly anaphylactic shock, though rare, can pose a serious threat to patients’ lives. Beyond PD-1 inhibitors like camrelizumab, there is also a report concerning cadonilimab, a PD-1/CTLA-4 bispecific antibody developed by a Chinese company. This report introduced seven cases of infusion reactions caused by cadonilimab, with symptoms including chills, fever, and rash, even including blood pressure drop. After antiallergic treatment, three of these cases also underwent rechallenge with cadonilimab and successfully continued cadonilimab treatment without allergic reactions. Therefore, rechallenge after severe infusion reactions can also be attempted in some patients (12).

Additionally, there is a report of a patient with hepatocellular carcinoma treated with atezolizumab who experienced a severe allergic reaction, including blood pressure drop, oxygen saturation decrease, and loss of consciousness, only after being rescued in the ICU was the patient out of danger. Due to the good tumor treatment effect, the patient eventually chose to try the drug again. Through antihistamine, glucocorticoid, and other antiallergic drugs for pretreatment, and gradually increasing drug concentration and infusion rate for desensitization treatment, atezolizumab was eventually used again in the patient. In our report, the patient did not undergo desensitization treatment with gradually increasing drug concentration and infusion rate for subsequent treatments; if used in the future, such desensitization treatment may be safer.

**Figure 2:**
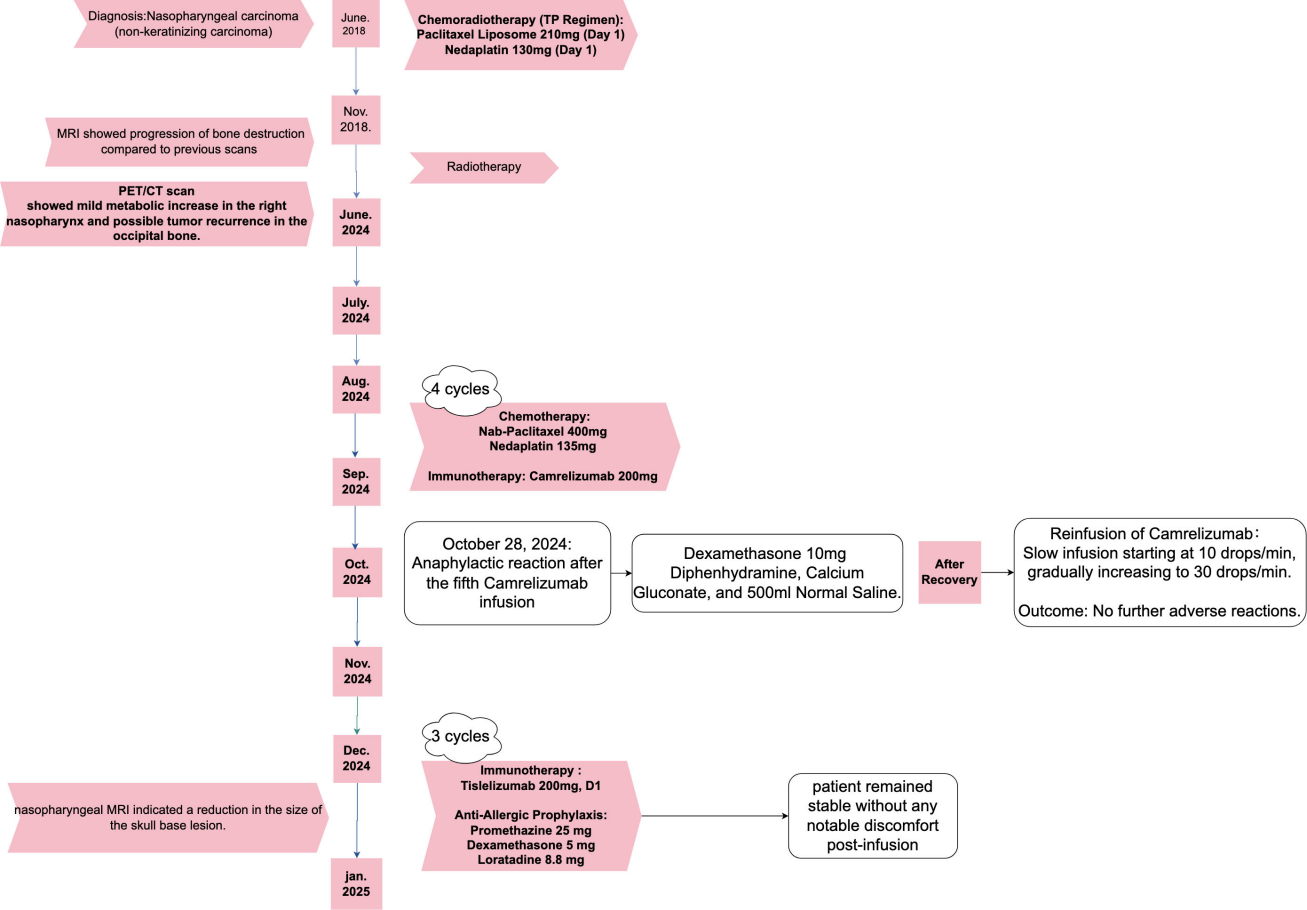
Timeline of the patient's treatment.

In this case, we observed that the patient developed an allergic reaction after the fifth cycle of camrelizumab treatment(as shown in **Figure 2**, By Figdraw.). Based on the timing of the reaction, we believe that this allergic response may be a pseudoallergic reaction rather than a typical IgE-mediated allergic reaction. According to the study by McNeil et al., MrgprX2 is a receptor almost exclusively expressed on mast cells, and it has been shown to cause mast cell activation in response to several chemotherapeutic agents (13, 14). The activation of MrgprX2 is not IgE-mediated but occurs through direct binding with the drug, leading to the release of mediators such as histamine from mast cells, thereby causing allergy-like symptoms. This finding is important for understanding allergic reactions induced by immunotherapeutic drugs, such as camrelizumab.

Furthermore, considering the low affinity characteristic of the MrgprX2 receptor, we hypothesize that the patient was able to tolerate rechallenge therapy by avoiding the rapid activation of the allergic threshold, thus successfully enduring the retreatment. Unlike IgE-mediated reactions, if the allergic reaction were IgE-mediated, rechallenge would typically not be successful, as IgE antibodies would quickly trigger a strong allergic response.

Therefore, considering the pseudoallergic reaction mechanism mediated by MrgprX2 is of significant value for the clinical application and management of future immunotherapies (15). With the development of MrgprX2 antagonists, therapeutic strategies targeting these non-IgE-mediated allergic reactions may offer more options for patients, especially for those who cannot tolerate conventional treatments.

In summary, although the incidence of acute allergic shock caused by camrelizumab is low, they seriously threaten patients’ lives, interrupt the continuity of immunotherapy, and affect the prognosis of tumor patients. With the increasing application of immunotherapy in clinical practice, allergy history and other risk factors should be carefully considered to minimize the occurrence of adverse reactions (16). At the same time, identifying factors related to anaphylactic shock caused by ICIs, screening susceptible patients, and clinical skin testing to reduce the risk of anaphylactic shock are issues that deserve attention and in-depth research.

## Data Availability

The original contributions presented in the study are included in the article/supplementary material. Further inquiries can be directed to the corresponding author/s.
